# Comparative analysis of linker histone H1, MeCP2, and HMGD1 on nucleosome stability and target site accessibility

**DOI:** 10.1038/srep33186

**Published:** 2016-09-14

**Authors:** Caitlyn Riedmann, Yvonne N. Fondufe-Mittendorf

**Affiliations:** 1 Department of Molecular and Cellular Biochemistry, University of Kentucky, Lexington, KY 40536, USA

## Abstract

Chromatin architectural proteins (CAPs) bind the entry/exit DNA of nucleosomes and linker DNA to form higher order chromatin structures with distinct transcriptional outcomes. How CAPs mediate nucleosome dynamics is not well understood. We hypothesize that CAPs regulate DNA target site accessibility through alteration of the rate of spontaneous dissociation of DNA from nucleosomes. We investigated the effects of histone H1, high mobility group D1 (HMGD1), and methyl CpG binding protein 2 (MeCP2), on the biophysical properties of nucleosomes and chromatin. We show that MeCP2, like the repressive histone H1, traps the nucleosome in a more compact mononucleosome structure. Furthermore, histone H1 and MeCP2 hinder model transcription factor Gal4 from binding to its cognate DNA site within the nucleosomal DNA. These results demonstrate that MeCP2 behaves like a repressor even in the absence of methylation. Additionally, MeCP2 behaves similarly to histone H1 and HMGD1 in creating a higher-order chromatin structure, which is susceptible to chromatin remodeling by ISWI. Overall, we show that CAP binding results in unique changes to nucleosome structure and dynamics.

Eukaryotic genomic DNA compacted into chromatin, a DNA-protein complex, results in DNA binding proteins being occluded from their target sites^1^,^2^,^3^. By virtue of physically blocking access of regulatory proteins, chromatin regulates cellular processes that require DNA as a substrate^4^,^5^,^6^. Although ATP-dependent remodeling can account for changes in chromatin structure, these changes are unable to explain how ATP-dependent DNA remodeling proteins recognize and access their target sites buried within chromatin. Chromatin structure is dynamic and its basic unit, the nucleosome, undergoes spontaneous fluctuations, termed ‘nucleosome breathing’^7^,^8^. This breathing results in the transient dissociation of DNA from the histone octamer allowing target sites of regulatory proteins normally occluded by nucleosomes to become intermittently accessible to their DNA binding proteins^3^,^9^,^10^,^11^. While the effect of factors such as histone post-translational modifications (PTMs) on nucleosome breathing have been extensively studied^8^,^12^,^13^,^14^,^15^,^16^,^17^, the effect of chromatin architectural proteins (CAPs) on nucleosome breathing has remained generally unexplored. We hypothesize that by binding the entry/exit DNA and the nucleosome dyad^18^,^19^,^20^, CAPs alter the rate of nucleosome breathing, thereby adding another level of regulation on essential biological functions.

CAPs are essential for maintaining cellular homeostasis as demonstrated by the fact that their expression levels are tightly controlled and CAP ratios have cell and tissue type specificity^18^,^21^,^22^,^23^,^24^. For example, mature neurons express the CAPs linker histone H1 and methyl CpG binding protein 2 (MeCP2) at a 1:1 ratio^24^, a ratio unique to mature neurons. Most tellingly, the mature neurons of patients with Rett Syndrome (RTT), who have loss of function mutations in MeCP2 can also be distinguished by their altered expression levels of histone H1 (and the corresponding change in ratio between the CAPs), coupled with widespread transcriptional deregulation^24^,^25^.

Nucleosome stability is frequently regulated through changes in their composition (e.g. DNA sequence, PTMs, and histone variants). Studies on the effect of CAPs on nucleosome stability and dynamics are limited. For instance, a few studies have been carried out to determine the effect of histone H1 on nucleosome dynamics^26^, or the comparative effect of histone H1 and either HMGD1^17^ or MeCP2^27^ on nucleosome dynamics. Although these studies revealed and quantified equilibrium aspects of the underlying behavior of CAPs and nucleosomes, no direct comparative measurements have been made on the effect of histone H1, MeCP2, and HMGD1 on spontaneous DNA unwrapping or rewrapping. Here, we have used *in vitro* reconstitution to measure the biophysical properties of CAP-bound nucleosomes to reveal the mechanisms underlying the influence of CAPs on nucleosome and chromatin dynamics. For these studies, we chose the CAPs linker histone H1 (well-documented in gene repression)^19^,^20^,^28^, HMGD1 (involved in gene activation)^20^,^29^,^30^, and the neural CAP MeCP2 (which activates and represses transcription in mature neurons)^20^,^22^,^24^,^30^,^31^,^32^,^33^,^34^. We carried out a series of biophysical and biochemical experiments to determine how these CAPs regulate nucleosome breathing dynamics and DNA target site accessibility in mononucleosomes and higher-order chromatin structures. We demonstrate that the binding of histone H1 or MeCP2 traps the mononucleosome in a wrapped state and reduces DNA target site accessibility. Binding of the activating CAP HMGD1 does not seem to affect FRET efficiency in this system. Interestingly, all three CAPs mediated the formation of a higher-order chromatin structure that is susceptible to chromatin remodeling. Our data sheds light on the *in vivo* requirements for CAP mediated changes in chromatin structure needed for DNA-templated cellular processes.

## Results

### CAPs have unique binding properties to nucleosomes

**Figure 1:**
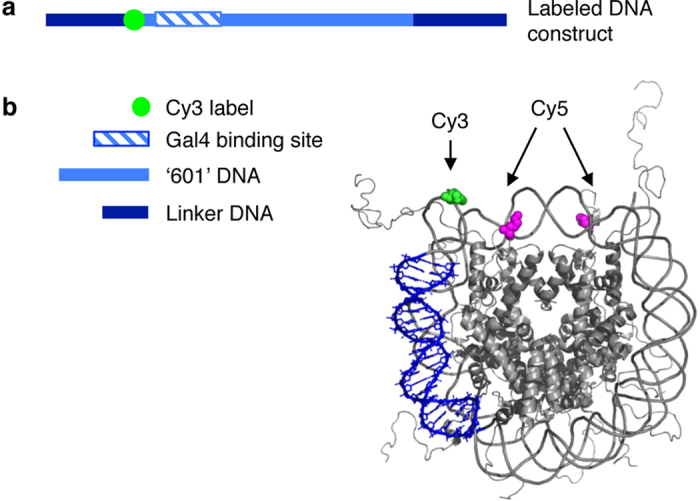
Nucleosomal construct used in FRET study (**a**) DNA construct used in our FRET studies^46^; contains a 601 NPS, the Cy3 fluorophore (position 43), a Gal4 binding site shown as a blue bar (position 48–76), and an extra 50 bp linker DNA at the entry/exit ends of the 601 NPS. (**b**) Structure of Cy3-Cy5 labeled nucleosome construct adapted from^7^,^8^, showing the labels in relation to the DNA and histone octamer.

**Figure 2:**
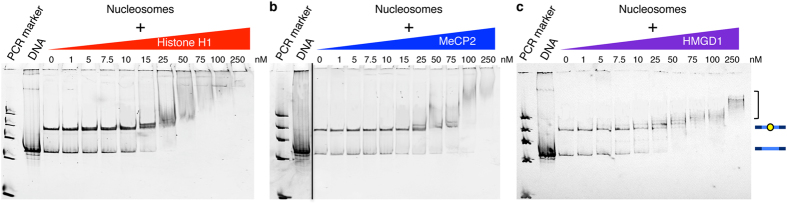
CAP-nucleosome binding. CAP-chromatosomes were created by titrating 0–250 nM of (**a**) histone H1, (**b**) MeCP2, or (**c**) HMGD1 into 5 nM labeled nucleosomes. Once binding equilibrium was reached, samples were run on a 5% native acrylamide gel to view CAP-chromatosome formation.

To understand the effect of histone H1, MeCP2 and HMGD1 on nucleosomes, we first determined if they bind to mononucleosomes with different affinities. Purified recombinant CAPs (Supplemental Fig. 1) were titrated into a fixed concentration of purified mononucleosomes (5 nM) (Supplemental Fig. 2a) with 24 base pair (bp) linker DNA on each side of the 601 nucleosome positioning sequence (NPS)^35^ (Fig. 1a–c). *In vitro* nucleosome purification typically results in a heterogeneous population of nucleosomes (centered and off-centered positions), even when assembled on an NPS sequence^36^. To ensure homogeneity of nucleosomes, we heated the samples at 55 °C for two hours, which resulted in the most favorable centered position (Supplemental Fig. 2). Efficiency of CAP-specific chromatosome assembly was monitored by nucleoprotein acrylamide gel electrophoresis (Fig. 2a–c). We observed a concentration-dependent gel-shift, which differed among the three CAPS studied. *Drosophila* histone H1 caused a shift between 10 nM and 15 nM, seen by the shift in the nucleosome band, with a further shift occurring at 50 nM of histone H1 (Fig. 2a). Aggregation occurred when concentrations greater than 75 nM of histone H1 were added (Fig. 2a). Concentrations between 25 nM and 50 nM of MeCP2 shifted the nucleosomes, with a super shift occurring at 50 nM and above (Fig. 2b). No aggregates were visibly observed at the experimental concentrations (1 nM–250 nM) (Fig. 2b). A *Drosophila* HMGD1 concentration between 1 nM and 5 nM caused a gel-shift, with more laddering observed beginning at 10 nM, as evident with the bands just above the nucleosomal band (Fig. 2c). This step-wise shift of nucleosome binding by HMGD1 is consistent with previous studies^37^. Overall, these results demonstrate that under our experimental conditions, all three CAPs bind nucleosomes with similar, yet distinct, profiles.

### Histone H1 and MeCP2 binding modulates nucleosome breathing dynamics

Since CAPs bind to the entry/exit DNA of the nucleosome, we hypothesize that the binding of CAPs would stabilize either an open or closed nucleosome conformation. To test this, we determined how nucleosome breathing dynamics change in response to CAP binding. A previously described Cy3-Cy5 paired mononucleosome Förster resonance energy transfer (FRET) system was used^8^. The nucleosome construct, has a Cy3 labeled 601 NPS with 50 bp entry and exit linker DNA wrapped around a histone octamer with a Cy5 label on both copies of the H2A core histone (Fig. 1). Since FRET efficiency is a measure of the distance between the Cy3 label on the DNA and the Cy5 label on the histone octamer, changes in FRET efficiency can be attributed to the wrapping/unwrapping of the DNA from the octamer core or sliding. CAPs are not known to slide nucleosomes along the DNA, so any change in FRET efficiency is most likely due to a change in the wrapping/unwrapping of the DNA from the nucleosome. Thus, based on our hypothesis, we predict that repressive chromatosomes (nucleosomes bound by a repressive CAP) will stabilize the DNA around the histone octamer, trapping the nucleosome in a wrapped conformation thereby leading to an increase in FRET efficiency. On the other hand, activating chromatosomes will either have no effect on nucleosome breathing, resulting in no change in FRET signal, or will stabilize the opening of the nucleosome resulting in a decrease in FRET efficiency.

**Figure 3:**
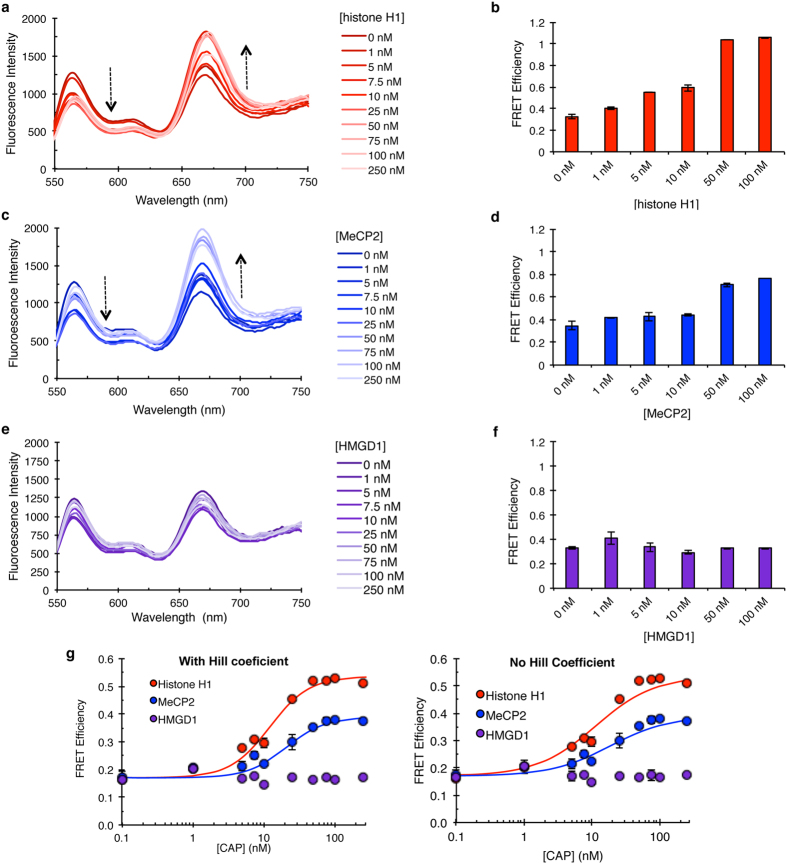
Histone H1 and MeCP2 influence nucleosome breathing. CAPs were titrated into 5 nM Cy3-Cy5 paired FRET mononucleosomes to measure changes in nucleosome breathing dynamics due to CAP binding. The CAP-chromatosome was excited with 510 nm light and the emission spectrum collected from 550–750 nm. FRET efficiency was calculated using the Ratio_A_ method^64^,^65^. Arrows indicate direction of changes in signal with Cy3 and Cy5. (**a**) Emission spectra and (**b**) the corresponding increase in FRET efficiency for select concentrations of the *Drosophila* histone H1 titration into labeled nucleosomes. (**c**) Emission spectra and (**d**) the corresponding changes in FRET efficiency for select concentrations of the MeCP2 titration into labeled nucleosomes. (**e**) Emission spectra and (**f**) the corresponding changes in FRET efficiency for select concentrations of HMGD1 titration into labeled nucleosomes. Normalized spectral graphs (to Cy3 emission) for select concentrations of CAPs are in Supplementary Fig. 3 to visualize the change in Cy5 emission. (**g**) The FRET efficiency measurements were plotted on a semi-log graph based on CAP concentration and fit to a Hill binding curve (shown are curves fitted with or without Hill coefficients). Due to the lack of measurable change in FRET efficiency upon HMGD1 binding, no Hill curve was drawn. Note: Error bars for all graphs are the standard deviation of 3 replicates.

To test this hypothesis, CAPs were titrated (1 nM–250 nM) into a fixed concentration (5 nM) of mononucleosomes. As expected, increasing the amount of the repressive CAP *Drosophila* histone H1 led to an increase in the wrapping of the nucleosome evidenced by an increase in FRET signals with correlating increase in efficiency (Fig. 3a,b, Supplemental Fig. 3a). These results are similar to our recent studies on human histone H1.0^26^. Similarly, increasing amounts of MeCP2 led to an increase in FRET efficiency (Fig. 3c,d, Supplemental Fig. 3b), suggesting that MeCP2 also traps the nucleosome in a wrapped conformation. In contrast, increasing amounts of the activating CAP HMGD1 did not change the FRET efficiency (Fig. 3e,f, Supplemental Fig. 3c).

We next calculated the affinities of the binding effect by fitting the FRET efficiency values to a Hill binding curve. Due to the lack of measurable change in FRET efficiency upon HMGD1-chromatosome assembly, only the data for histone H1 and MeCP2 chromatosome formation was analyzed (Fig. 3g). From these analyses, less Histone H1 is required to trap the nucleosome in a wrapped conformation than MeCP2, exhibiting an S_1/2_ value of 12.09 ± 2.08 nM for histone H1 compared to 20.73 ± 4.98 nM for MeCP2 (Table 1). These values correspond with the range of concentrations of histone H1 and MeCP2 needed to bring about a noticeable gel shift in our electromobility gel shift (EMSA) experiments (Fig. 2a,b). The observed S_1/2_ value for *Drosophila* histone H1 is similar to our recent data of human histone H1.0^26^. Furthermore, *Drosophila* histone H1 binding leads to a higher FRET efficiency compared to MeCP2 (Fig. 3g), indicating that histone H1 is able to stabilize a more highly wrapped nucleosome structure than the structure stabilized by MeCP2.

### Histone H1 and MeCP2 alter DNA target site accessibility within nucleosomes

**Figure 4:**
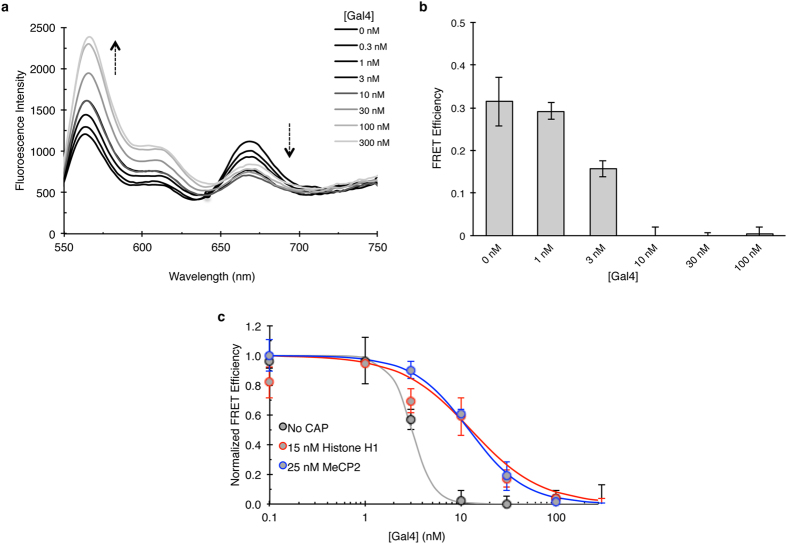
Histone H1- and MeCP2-chromatosomes hinder Gal4 from binding its target site within mononucleosomes. (**a**) Fluorescence emission spectra of Cy3-Cy5 labeled mononucleosomes with increasing concentrations of Gal4 (Normalized to Cy3 emission spectral graphs for select concentrations are in Supplemental Fig. 4). As Gal4 binds to its target site, the Cy5 emission decreases leading to a decrease in FRET efficiency (**b**). (**c**) The normalized FRET efficiency measurements (complete spectra graphs are in Supplemental Fig. 3) of a Gal4 titration into mononucleosomes not stabilized by CAP, bound with 15 nM histone H1, or 25 nM MeCP2 were plotted on a semi-log plot based on Gal4 concentration. Note: the concentrations for CAPs used, were based on their pre-determined S_1/2_ values (Table 1). Values represent averages of duplicate or triplicate experiments and error bars depict the standard deviations of the mean.

Due to the observed effects on the breathing dynamics of histone H1- and MeCP2- chromatosomes we asked if the binding of these CAPs change DNA target site accessibility. To address this, a Gal4 binding site was incorporated into positions 8–26 of the 601 NPS (equal to positions 58–76 bp of the full 247 bp construct) (Fig. 1a). The binding of the model transcription factor (TF) Gal4 to its target sequence traps the nucleosome in a partially unwrapped state and sterically prevents nucleosome rewrapping, thereby leading to a decrease in FRET efficiency^38^,^39^. We first confirmed the binding of Gal4 to our mononucleosomal construct and the subsequent reduction in FRET efficiency by titrating increasing amounts of Gal4 (0.1–300 nM) into 5 nM of mononucleosomes (Fig. 4a,b, Supplemental Fig. 4a). Based on our hypothesis, repressive chromatosomes will limit the spontaneous breathing of the nucleosome, thereby hindering Gal4 from binding its target site. This will result in a higher concentration of Gal4 required to decrease the FRET efficiency compared to a nucleosome that is not stabilized by repressive CAP binding. We tested this hypothesis in a dynamic environment by first trapping the nucleosomes in a wrapped state with 15 nM histone H1 or 25 nM MeCP2 (based on their respective S_1/2_ values, Table 1) before Gal4 was titrated into the sample (0.1–300 nM) (Supplemental Fig. 4b,c). We fit the FRET efficiency values to a Hill binding curve to determine the S_1/2_ concentration of Gal4 needed to open the nucleosome (Fig. 4c, Table 2). When the nucleosome was not CAP-bound (No CAP), the S_1/2_ value was 3.06 ± 0.14 nM Gal4 (Table 2). Consistent with our hypothesis, when the nucleosome was stabilized with *Drosophila* histone H1 (histone H1-chromatosome) the S_1/2_ value increased more than 4 fold to 12.75 ± 4.6 nM of Gal4. Similarly, MeCP2 chromatosomes required 12.16 ± 1.34 nM of Gal4 to drive the opening of the nucleosome (Table 2). These results demonstrate that both histone H1 and MeCP2 trap the nucleosome in a wrapped state, inhibiting Gal4 from binding to its target site. Furthermore, even in the presence of H1 and MeCP2, Gal4 is still able to bind within the nucleosome via DNA unwrapping, however both CAPs regulate TF binding by suppressing the probability of the nucleosome being partially unwrapped.

### Higher-order chromatin structures mediated by CAPs are subject to chromatin remodeling

In cells, nucleosomes are found within the context of a higher order chromatin structure dictated by CAPs^18^,^20^. We therefore asked how the higher-order chromatin structure specified by the CAPs of interest affected DNA target site accessibility. We used a previously described system whereby histone octamer is reconstituted with a 3 kilobase (kb) DNA array template containing a 17-mer of the 601 NPS separated by 30 bp of linker DNA^40^ following previous protocols (Supplemental Fig. 5a)^41^,^42^. Excess protein and buffering DNA was removed through a 5–40% sucrose gradient purification, retaining the CAP-specific chromatin structures (Supplemental Fig. 5b,c). We subjected each array preparation to the described quality controls to assure nucleosome occupancy. We controlled for the proper positioning and occupancy of the 601 sequences by histone octamers and CAPs by digesting the arrays with AvaI, which cuts within the linker DNA between the 601 NPSs, (Supplemental Fig. 5d). The ratio of mononucleosomal DNA to free DNA serves as a measure of the degree of saturation. The control template (No CAP) and the CAP arrays were equally saturated with octamers (Supplemental Fig. 5d lanes 2–5). The degree of saturation was calculated to be about 90% (No CAP-, histone H1-, MeCP2-, and HMGD1-arrays). We also validated that these were CAP-specific chromatosome arrays by running the chromatin arrays on an SDS-PAGE gel and staining with coomassie blue to confirm incorporation of the input CAPs and histones (Supplemental Fig. 5e). This scaled-down chromatin structure was subjected to a restriction enzyme accessibility assay^43^,^44^.

**Figure 5:**
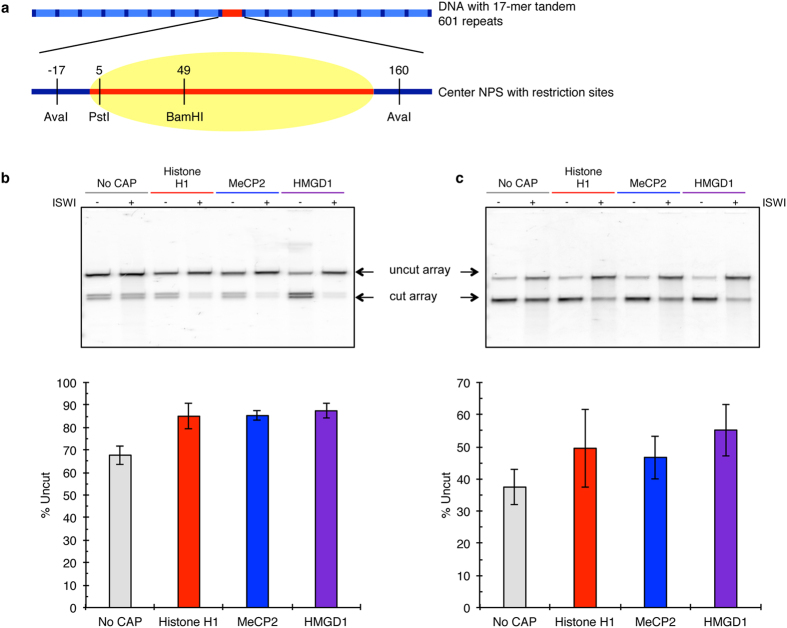
CAP-mediated higher order chromatin structures are susceptible to chromatin remodeling by ISWI. (**a**) Scheme of the 3055 bp DNA construct containing a 17-mer array of 601 NPS separated by 30 bp linker DNA. The array DNA comprised 17 repeats of a NPS harboring the Widom-601 nucleosome positioning sequence (dashed line). Numbers indicate positions of restriction enzyme sites with respect to the nucleosome boundary^46^. (**b**) Remodeled and non-remodeled (ISWI or no ISWI) chromatin samples were digested with BamHI. ImageQuant imaging software was used to measure the intensity of the full and digested array DNA. These values were used to determine the percent uncut. (**c**) After ISWI remodeling, chromatin samples were digested with PstI and imaged on an agarose gel. Digestion amount quantified using band intensity, accounting for non-saturated arrays and were compared to No CAP chromatin. Error bars are the mean ± standard deviation.

We tested for the accessibility of two restriction enzymes: BamHI and PstI, located at positions 5 and 49 respectively of the central NPS (Fig. 5a). Chromatin accessibility by these enzymes is measured by comparing the restriction enzyme-dependent decrease in band intensity of the full-length array DNA (3 kb) with a concomitant increase in the cleavage product (1.5 kb), which is half the length of the full array. Both restriction enzymes work under conditions containing Mg^2+^, which in itself compacts nucleosomal array *in vitro*. Our experiments were carried out in 1 mM Mg^2+^ and under this condition, nucleosomal arrays *in vitro* undergo a reversible compaction resembling compaction of native chromatin fibers *in vivo*^45^. Since this ionic environment is the same in all experiments, any observed change in digestion would be caused by a difference in chromatin structure induced by CAP binding.

For the BamHI digest, the arrays were all exposed to predetermined concentrations of BamH1 (40 units of BamHI for an hour). Digested arrays were deproteinized, ran on an agarose gel with subsequent quantification of the resultant bands. Our results show BamHI digested about 20% of the arrays (Fig. 5, Lanes 1, 3, 5 & 7). At first this was surprising based on our controls above with AvaI digests, which showed that >90% of the arrays were completely saturated (Supplemental Fig. 5d). On the other hand, it is known that during the processing of arrays about 10% of histones fall off the sequences^46^. Furthermore, salt dialysis *in vitro* reconstitutions result in irregularly positioned arrays, which can be remodeled by ISWI into regularly-positioned arrays^36^,^47^. We therefore asked if remodeling of these arrays by ISWI would ameliorate the differences in restriction enzyme digest. Interestingly, while BamHI digestion of No CAP array resulted in slight but not significant differences in digest before and after remodeling (Fig. 5b; Lanes 1 & 2). However when chromatosome arrays were remodeled with ISWI, we observed significant protection from BamHI digest (Fig. 5b, compare Lanes 3–4; 5–6; 7–8). These results suggest that CAP-dependent structures play a role in chromatin remodeling by ISWI, resulting in regularly spaced arrays. Our results are consistent with previous studies showing that ISWI regulates higher-order chromatin structure by promoting the assembly of H1-containing chromatin^48^,^49^.

Next, we tested the accessibility of PstI, whose site on the NPS, near the entry/exit site, is a better representation of a TF binding site *in vivo*^50^,^51^,^52^. Thus, we used the accessibility and digestion of the PstI site, located on 1–6 bp from the start of the NPS of the central nucleosome (Fig. 5a) as a measure of TF accessibility, similar to Gal4 in the mononucleosome studies (Fig. 4). Due to its proximity to the end of a nucleosome, we predict that this site will be more easily accessible and therefore be cleaved more. Since remodeling by ISWI results in a more regular chromatin structure, we tested the accessibility of PstI under remodeled conditions. PstI digestion in No CAP chromatin array was higher, as expected, while it was reduced in the CAP-chromatosome arrays (Fig. 5c). We did not observe any significant differences in PstI digest after remodeling among the three CAPs. We also show as a control that the presence of CAPs and ISWI did not prevent these restriction enzymes from digesting their sites on the array DNA (Supplemental Fig. 6). We conclude that ISWI remodels CAP-dependent structures containing physiological amounts of histone H1, MeCP2, and HMGD1.

## Discussion

Many aspects of chromatin structure, including the contributions of histone H1 and HMGD1, have been characterized; however, to date, the present study is the first comparative analysis of histone H1, MeCP2, and HMGD1 on nucleosome dynamics and DNA accessibility within nucleosomes and chromatin. These studies demonstrate that the binding of these three CAPs facilitate different mononucleosome ‘breathing’ and DNA target site accessibility profiles that imply their gene regulatory outcomes. The proteins studied share several nucleosome binding characteristics: they bind entry/exit and linker DNA, they compete for binding to this site, and they result in changes to the transcription^17^,^27^. Our studies clearly show that both *Drosophila* histone H1 and human MeCP2 traps the mononucleosome in a compact state and reduce target site accessibility. We also demonstrate that HMGD1 binding does not affect ensemble FRET efficiency.

MeCP2, named for its ability to bind methylated DNA, is present at about equal amounts to histone H1 in mature neurons^24^,^53^ and competes with histone H1 for nucleosome binding^54^,^55^. We found that similar to histone H1, MeCP2 stabilizes a closed mononucleosome conformation (Fig. 3), reduces the target accessibility of mononucleosomal DNA (Fig. 4), and reduces accessibility in chromatin arrays to restriction enzyme digestion at about an equal level to histone H1 (Fig. 5). How these proteins are recruited to particular chromatin regions is still unknown. It is possible that MeCP2 is recruited to particular nucleosomes by methylation, increasing its affinity for binding, and therefore becoming an even more potent repressor. However, future studies are needed to verify this idea. HMGD1 on the other hand, had no measurable effect on the dynamics of a breathing nucleosome as measured by FRET (Fig. 3). Since these are *in vitro* measurements, it is possible that *in vivo*, the interplay of CAPs with other epigenetic factors is required for robust chromatin changes in DNA accessibility.

### Chromatin remodeling in the presence of CAPs

Studies have shown that in the absence of histone H1, chromatin folds into irregular arrays, rather than into the more highly-ordered 30-nanometer fibers characteristic of native chromatin^56^,^57^. These observations imply that CAPs contribute to the free energy of chromatin folding, and that they help select a single folded state from among the set of compact states that may be reached in its absence. Our data shows that in the presence of remodelers, CAPs contribute to stabilizing a regular array structure (Fig. 5b). Thus, it is possible that by biasing nucleosome positioning, CAPs could modulate the accessibility of nucleosomal DNA sites. Alternatively, by binding to DNA at the entry/exit sites of the nucleosome, CAPs could modulate the spontaneous unwrapping of DNA from the nucleosome or alter the fraction of time that the DNA is unwrapped and accessible (Fig. 4). Previous results have also shown that the addition of histone H1 to chromatin can switch SWI/SNF remodeling from favoring end positions to favoring central positions^58^. Thus, the results presented here indicate that histone H1, MeCP2, and HMGD1 can also influence the specificity of nucleosome repositioning (Fig. 5). In contrast, a recent study showed that HMGB1 (a human HMGD1 homolog) increased the rate of repositioning by the ISWI ATPase or two complexes based on it (ATP-utilizing Chromatin Assembly and Remodeling Factor [ACF] and Chromatin Accessibility Complex [CHRAC]), but did not change the resulting octamer location^59^. Taken together, these observations suggest that different chromatin associated proteins might regulate transcription by differentially modulating chromatin remodeling complex action and our data suggest that CAPs could also be playing a role.

### Biological consequences of histone the incorporation of histone H1, MeCP2, and HMGD1 into chromatin

During early development in higher eukaryotes, HMG1 is the only CAP present, allowing for rapid replication and transcription of genes^60^. As development progresses, histone H1 expression increases resulting in functional, specialized chromatin domains necessary for cell type-specific gene expression. While most cells and organisms have between 0.5 and 1 histone H1 protein per nucleosome^61^, the amount of CAPs differ among cell types and developmental stages^61^,^62^,^63^. One possible explanation for the changes in relative abundance of CAPs and the corresponding transcriptional changes necessary throughout development and differentiation is that CAPs are regulated through an equilibrium mechanism in which final transcriptional activity depends not only on the abundance and affinity of transcription factors, but also on the abundance and affinity of CAPs. Since CAPs compete with each other for nucleosome binding, the interplay between relative CAP concentrations may determine which CAP is localized to the chromatin at any given genomic region *in vivo*. This may not be as simple as it sounds. For instance, in histone H1 knockdown cells we observed HMGD1 occupying previously bound histone H1 promoters, with no correlation in increased gene expression^20^. On the other hand, in HMGD1 knockdown cells, previously bound HMGD1 promoters became occupied by histone H1, with a consequent decrease in gene expression^20^. We hypothesize that *in vivo* the relationship between CAPs and transcriptional outcome is not straightforward, as other epigenetic factors can influence these effects, either by reducing or enhancing the consequences of CAP binding. Since CAPs are predicted to assemble a distinct higher-order chromatin structure, genomic chromatin will not be structurally homogeneous, but rather, partitioned into many different micro-domains dictated by the specific protein composition of the region in question. Thus, the presence of CAPs may be essential in providing the functional specialization of chromatin domains within different cell-types and lineages.

In these studies, we determined the effect of histone H1, MeCP2, and HMGD1 on nucleosome and chromatin structures to show the unique structural contributions provided by each CAP. Our *in vitro* data sheds light on the *in vivo* alterations in chromatin structure produced by CAPs and provides a way to ascertain the nuanced differences in CAP binding to nucleosomes/chromatin and the DNA accessibility within these chromatosome structures. Such studies contribute critical information for understanding the epigenetic roles played by CAPs, histone PTMs, and DNA methylation in the fine-tuning of chromatin mediated gene regulation within the cell.

## Materials and Methods

### Protein purification

*Drosophila histone* H1, HMGD1, and human MeCP2 cDNAs were amplified and cloned into the Champion pET SUMO system (Invitrogen) for protein expression. Studies were carried out according to manufacturer instructions. Briefly, the cell pellet was resuspended in Buffer A (25 mM Tris-HCl pH 8.0, 150 mM NaCl, 1 mM PMSF, and 5 mM Imidazole) and broken with a French press. The crude homogenate was centrifuged at 30,000 × g for 45 minutes at 4 °C, and the supernatant filtered through a 10 ml nickel column (His Select resin from Sigma) by gravity. The column was washed twice with 100 ml of Buffer B (NaCl in 25 mM Tris-HCl pH 8.0) and then with Buffer A2 (buffer A with 0 mM PMSF) until the wash fraction exhibited an OD280 of 0.02 or less. Bound proteins were then eluted with 125 mM imidazole. The His^6^-SUMO tag was removed from the proteins through overnight digestion at 4 °C with SUMO protease and dialyzed against buffer A2. HMGD1 was further purified on a HiTrap Heparin HP column (GE Healthcare) with a 0–1 M NaCl gradient (eluted at ~400 mM NaCl). Histone H1 and MeCP2 were further purified on a Mono S cation exchange column (GE Healthcare) using a 150 mM–800 mM NaCl gradient (histone H1 and MeCP2 eluted at ~600 mM NaCl). MeCP2 was further purified on a chitin column (NEB). MeCP2 was washed with a 1 M NaCl solution, and eluted with 50 mM DTT, 200 mM NaCl in potassium phosphate buffer, pH 7.5. Amicon Ultra centrifugal filters (Millipore) were used to buffer exchange and concentrate the proteins to ~0.5–1 mg/ml. Histone H1 was stored in a 200 mM NaCl solution, MeCP2 in a 500 mM NaCl solution, and HMGD1 in a 200 mM NaCl solution (for protein stability).

### Histone octamer labeling

H2A (K119C) histone octamer was labeled with Cy5-maleamide (GE Healthcare) as previously described^15^, with labeling efficiency of 85%. Briefly, lyophilized histones were unfolded in unfolding buffer (7 M Guandinium, 10 mM DTT) at a concentration of 5 mg/mL for 1–3 h, and then spun to remove aggregates. Absorption at 276 nm was measured for each unfolded histone to determine concentration. Histones were combined in a ratio of H2A:H2B:H3:H4 of 1.2:1.2:1:1 then refolded by double dialysis into refolding buffer. The refolded octamer was labeled with Cy5 maleamide chemistry as previously described^15^, then purified over a Superdex 200 (GE Healthcare) gel filtration column to remove excess dimer, tetramer, and dye. The purity of each octamer was confirmed by SDS-PAGE and labeling efficiency determined by UV-VIS.

### Gel shift assays

CAPs (0–250 nM) were incubated with 5 nM mononucleosomes (pre-heated at 55 °C for 2 h) in 130 mM NaCl, 10% glycerol, 10 mM Tris HCl pH 8, and 0.005% Tween 20 in 0.5× TE (5 nM and 0.5 mM EDTA pH 8.0) at room temperature for 3 min. The mixture was loaded onto a pre-run 5% native polyacrylamide gel in 0.3× TTE (Tris-Taurine-EDTA) and electrophoresed at 300 V for 2 h.

### FRET measurements and efficiency analysis

Reactions (20 μl) containing 5 nM Cy3-Cy5 labeled nucleosomes, CAP, and/or Gal4 in 130 mM NaCl, 10% glycerol, 10 mM Tris HCl pH 8, and 0.005% Tween 20 in 0.5× TE were excited at 510 nm (Cy3-donor) and 610 nm (Cy5-acceptor), and the emission spectra was measured from 530–750 nm and 630–750 nm respectively using a Fluoromax-4 (Horiba) photon-counting steady state fluorometer at room temperature. MATLAB R2013b was used to integrate the donor emission data (656–674 nm) for both direct excitation and excitation due to energy transfer to determine the FRET efficiency using the Ratio_A_ method^64^,^65^. All measurements were taken in duplicate or triplicate and standard deviation used as a measure of uncertainty.

### Protein affinity determination

CAP binding was detected by measuring the change in FRET efficiency: histone H1 and MeCP2 produced an increase; Gal4 binding led to a decrease. SigmaPlot Version 13.0 was used to fit the FRET efficiency as a function of protein concentration to a Hill binding curve Y = E_min_ + (E_max_ − E_min_)/(1 + ([titrant]/S_1/2_) ^ (-Hill coefficient)). E_min_ is the minimum FRET efficiency measured, E_max_ is the maximum FRET efficiency measured, [titrant] is the concentration of titrant in nM, S_1/2_ is the concentration of titrant needed to bring about half the change in FRET efficiency (in nM).

### *In vitro* chromatin and chromatosome array reconstitution and purification

Mononucleosome and 17-mer arrays were formed by combining DNA and purified proteins in 2 M NaCl and 1 mM BZA in 0.5× TE and placed in double dialysis against 1 mM BZA in 0.5× TE at 4 °C (first dialysis for a minimum of 6 h, second dialysis overnight)^42^. Reconstituted chromatin was purified from excess reactants (free DNA/protein) using sucrose gradients in a Beckman Optima XPN-90 Ultracentrifuge. The fractions containing the desired product were pooled and a 30,000 NMWL Amicon Ultra centrifugal filter (Millipore) was used to remove residual sucrose and concentrate the chromatin to desired concentration. For mononucleosomal reconstitution: PCR amplified DNA containing the 601 sequence and linker was added at a 0.85:1 molar ratio to human histone octamer. After salt dialysis the reconstituted mononucleosomes were purified on a 5–30% sucrose gradient centrifuged at 41,000 × rpm for 22 h. For 17-mer chromatin/chromatosome array: human histone octamer (a kind gift from the Luger laboratory) and specified CAP was added to DdeI (NEB) digested 17-mer plasmid at a stoichiometry of 23(CAP):23(histone octamer):1(17-mer array). The DdeI digested plasmid^43^ contains the 3,055 bp array DNA, generating a 17-mer tandem repeat of the 601 NPS separated by 30 bp linker DNA and seven shorter DNA fragments, which serve as buffering DNA aiding in minimizing non-specific aggregation of the arrays^43^ of lengths 653, 535, 421, 404, 245, 230, and 161 (Supplemental Fig. 5). Reconstituted chromatin was loaded onto a sucrose gradient % and centrifuges for 8 hours at 39,000 rpm as above.

### Quality control of higher-order chromatosome arrays

#### Coomassie staining to characterize the protein content within the arrays

Purified chromatin and chromatosome arrays were precipitated with 5 mM MgCl_2_^66^ and heated at 90 °C in 1× SDS loading buffer and separated an 18% SDS denaturing polyacrylamide gel. The gel was stained with coomassie blue and the band intensities were measured using the Odyssey Infra Red Imaging system (Li-Cor).

#### AvaI digests to characterize the arrays

Purified chromatin and chromatosome arrays (100 ng based on their DNA content at 260 nm) were digested into monomers with 15 U AvaI (NEB) at 37 °C in 50 mM Potassium Acetate, 20 mM Tris-acetate, 10 mM Magnesium Acetate, and 1 mM DTT at pH 7.9 (20 μl final volume). The reactions were run on a 5% native polyacrylamide gel in 0.5× TBE (Tris-Borate-EDTA). Arrays had more than 90% saturation as measured by the comparison of the 200 bp fragment (naked DNA) and the ~400 bp fragment (nucleosomal fragment)^67^.

### Restriction enzyme accessibility assay

The restriction enzyme accessibility assay was performed with some modification as described^44^,^68^. 100 ng of the chromatin array (based on DNA content) was digested with 40 U of BamHI (NEB) or 5 U of PstI (NEB) for 1 h or 15 mins respectively at 37 °C in 100 mM NaCl, 50 mM Tris-HCl pH 8.0, 1 mM DTT, and 1 mM MgCl_2_. Reactions were stopped with 1.5 mM EDTA pH 8.0 followed by Proteinase K digestion for 1 h at 37 °C^43^. The DNA was run on a 1% agarose gel with 0.2× GelStar in 1× TAE (Tris-Acetic Acid-EDTA) at 100 volts for 1 h.

### Chromatin remodeling reactions

For probing nucleosomal array accessibility, 25 ng of chromatin or chromatosome arrays (based on DNA) were incubated with +/−300 nM of ISWI and 2 mM ATP in 100 mM NaCl, 50 mM Tris-HCl pH 8.0, 1 mM DTT, and 1 mM MgCl_2_ for 1 h at 27 °C, followed by adding either 40 U of BamHI (NEB) or PstI (NEB) and allowed to digest for 1 h or 15 min respectively at 37 °C. Reactions were stopped with 1.5 mM EDTA, followed by Proteinase K digestion for 1 h at 37 °C. Samples were electrophoresed on a 1% agarose gel with 0.2× GelStar in 1× TAE at 100 volts for 1 h.

### Gel staining and imaging

All gels were stained with GelStar (Lonza) and imaged on a Typhoon FLA 9500 from GE Healthcare using the ECF filter. Band intensities were quantified using ImageQuant.

## Additional Information

**How to cite this article**: Riedmann, C. and Fondufe-Mittendorf, Y. N. Comparative analysis of linker histone H1, MeCP2, and HMGD1 on nucleosome stability and target site accessibility. *Sci. Rep.* **6**, 33186; doi: 10.1038/srep33186 (2016).

## Supplementary Material

Supplementary Information

## Figures and Tables

**Table 1:** S_1/2_ values for the binding of CAPs to mononucleosomes.

CAP	S_1/2_ (nM)	Hill Coefficient
Histone H1	12.09 ± 2.08	1.55 ± 0.38
MeCP2	20.73 ± 4.98	1.59 ± 0.46
HMGD1	N/A	N/A

FRET efficiency values, with standard deviation from duplicate experiments, were fit to a Hill binding curve to determine S_1/2_ value of CAP binding.

**Table 2:** The S_1/2_ values of Gal4 binding to nucleosomes not stabilized by CAP, stabilized by 15 nM histone H1, or stabilized by 25 nM MeCP2.

Chromatosome	S_1/2_ (nM Gal4)	Hill Coefficient
No CAP	3.06 ± 0.14	−3.76 ± 2.16
15 nM Histone H1	12.75 ± 4.60	−1.18 ± 0.48
25 nM MeCP2	12.16 ± 1.34	−1.47 ± 0.22

FRET efficiency values, with standard deviation from duplicate or triplicate experiments, were fit to a Hill binding curve to determine the S_1/2_ value for Gal4 binding to nucleosomes not bound by CAP or bound by histone H1 or MeCP2.
