# Supplementary material for: Comparative analysis of linker histone H1, MeCP2, and HMGD1 on nucleosome stability and target site accessibility
Source: Sci Rep. 2016 Sep 14;6:33186. doi: 10.1038/srep33186 (PMC5021983; doi:10.1038/srep33186)
Supplement: Supplementary Information [file srep33186-s1.pdf]

Comparative analysis of linker histone H1, MeCP2, and HMGD1 on nucleosome stability and target site accessibility

Caitlyn Riedmann[1] and Yvonne Fondufe-Mittendorf[1*]

[1]Department of Molecular and Cellular Biochemistry, University of Kentucky, Lexington, Kentucky 40536

Supplemental Information

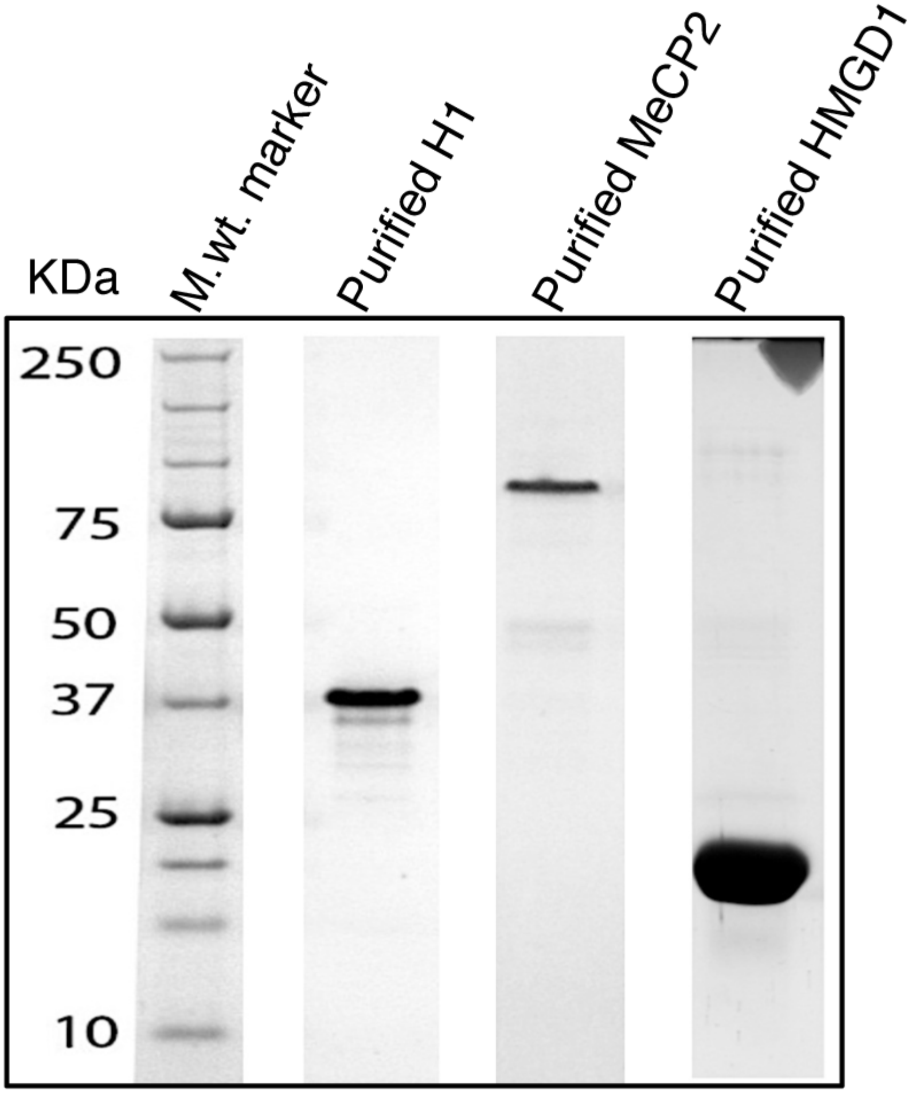

Supplemental Figure 1.

Supplemental Figure 1: Purification of recombinant CAP proteins. Coomassie blue staining of SDS denaturing protein gels of the purified recombinant CAPs used in all experiments.

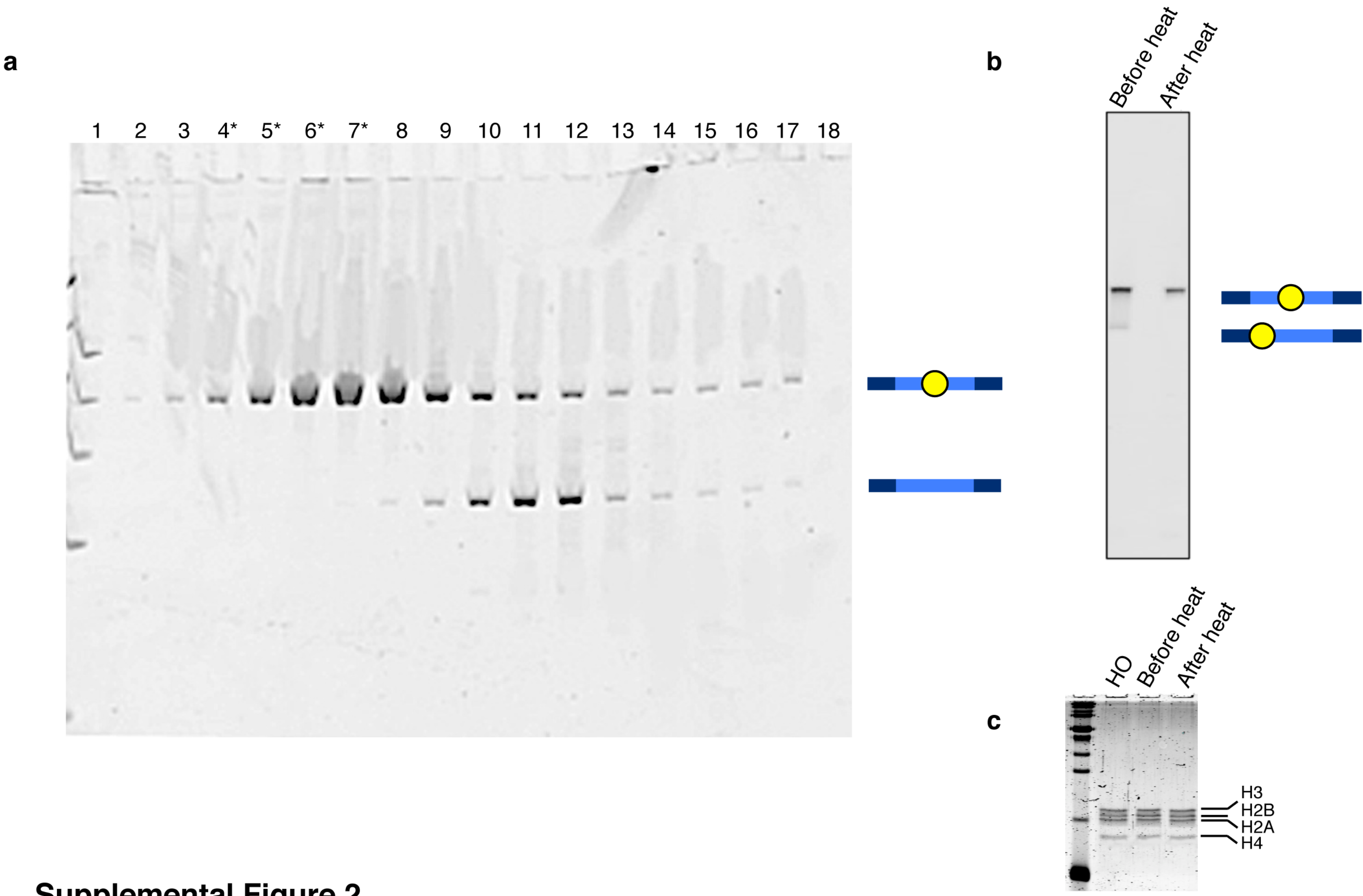

Supplemental Figure 2

Supplemental Figure 2: Purification of nucleosome. (a) Nucleosomal DNA and nucleosomes sediment differently on a 5-40 % sucrose gradient and can be purified using a 5 % native-PAGE. **(b)** After purification nucleosomes are heterogeneous (histone octamers centered and off-centered on the DNA). Heating nucleosomes at 55 °C for 2 hours centers the nucleosome. Shown below the DNA gels is a representative coomasie stained proetin gel showing the corresponding histone proteins. *fractions collected and pooled. Yellow circles represent the location of nucleosome on the DNA.

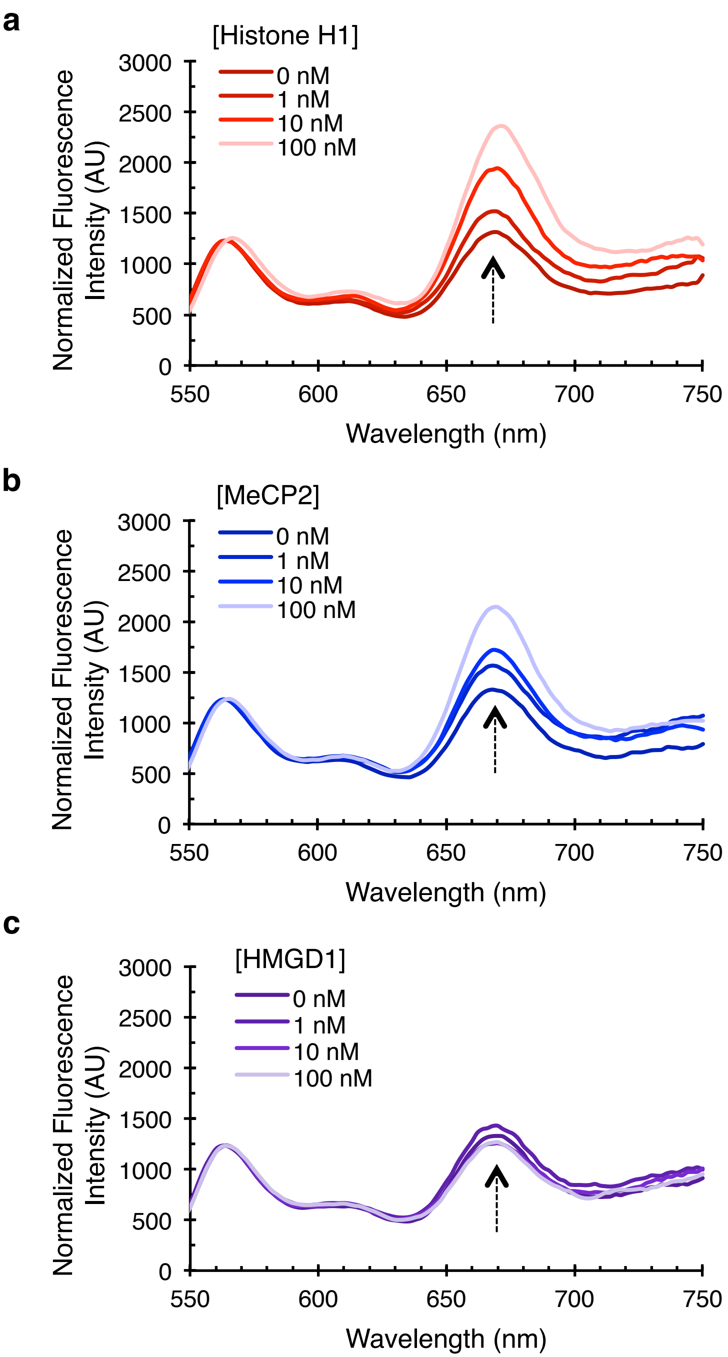

Supplemental Figure 3

Supplemental Figure 3: FRET spectra of CAP-chromatosomes. CAPs were titrated into 5 nM Cy3-Cy5 paired FRET mononucleosomes. The CAP-chromatosome was excited with 510 nm light and the emission spectrum collected from 550-750 nm for histone H1 **(a),** MeCP2 **(b)**, and HMGD1 **(c)**. Emission profiles were normalized to Cy3 emission values in order to more clearly see the change in Cy5 emission due to changes in FRET efficiency.

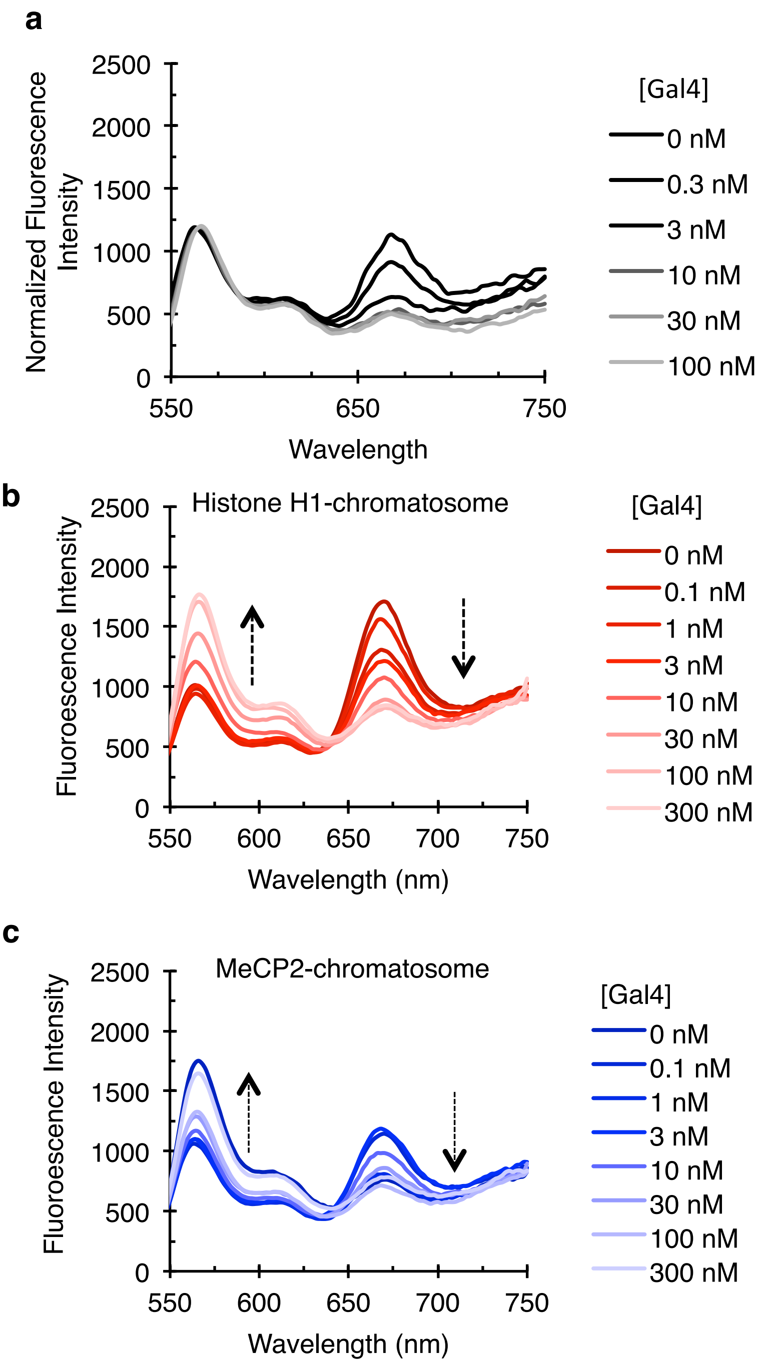

Supplemental Figure 4

Supplemental Figure 4: Gal4 binding traps mononucleosomes in an open conformation. Gal4 was titrated into 5 nM Cy3-Cy5 paired FRET mononucleosomes **(a)** not stabilized by CAPs bound by **(b)** 15 nM histone H1 or **(c)** 25 nM MeCP2. The complex was excited with 510 nm light and the emission spectrum collected from 550-750 nm.

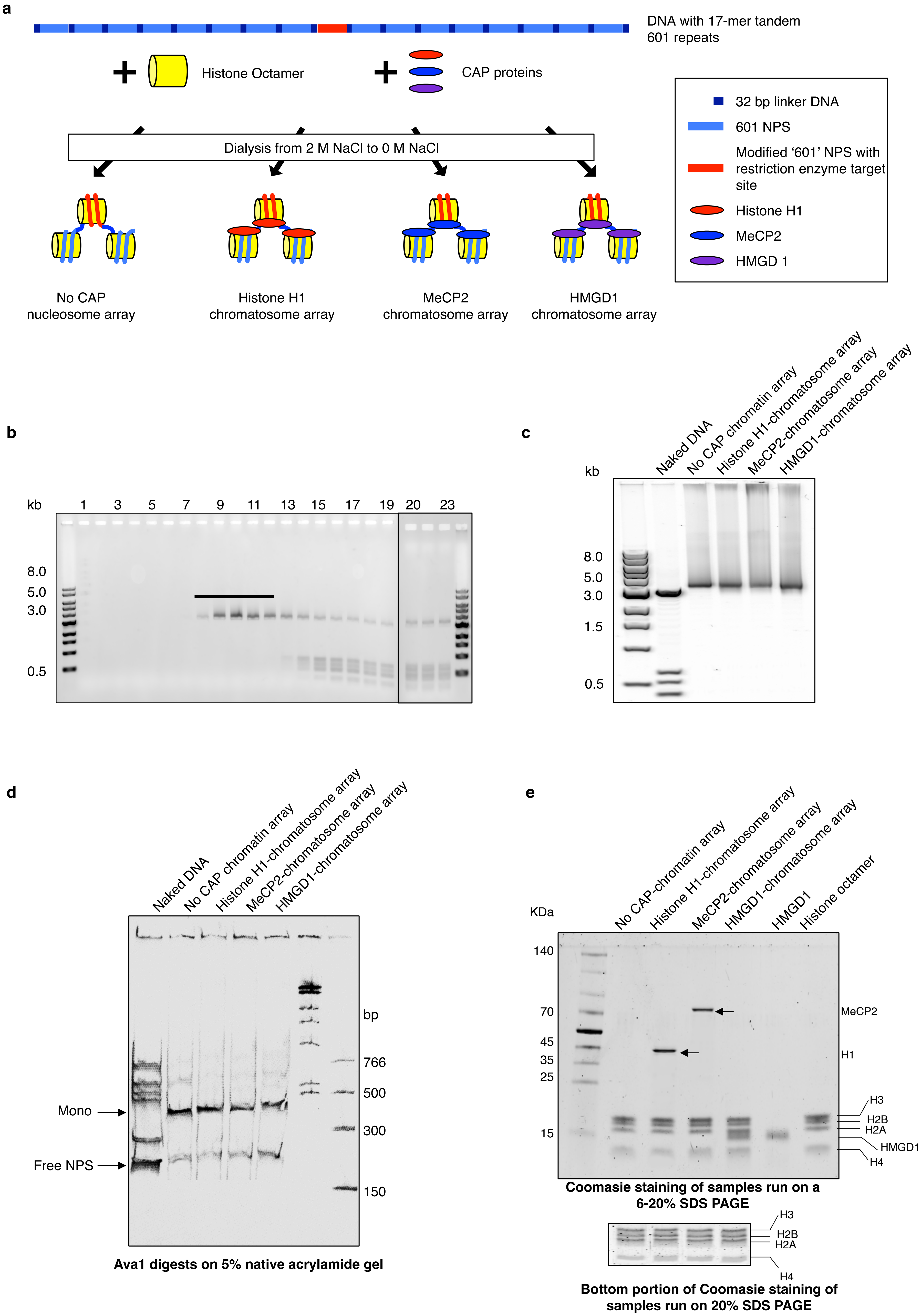

Supplemental Figure 5

Supplemental Figure 5: CAP-dependent higher order chromatin structures. (a) Schematic representation of the formation of the CAP-dependent structures, the 3055 bp array DNA plus buffering DNA (seven shorter DNA fragments of lengths 653, 535, 421, 404, 245, 230 and 161bp) was added to human histone core octamers and CAPs in 2 M NaCl. Through dialysis, the salt concentration was reduced to a no NaCl environment (0.5 x TE). (b) Fully reconstituted chromatin and chromatosome arrays were purified from buffering DNA and free proteins via a 5-40 % sucrose gradient (shown is a representative gel). Line shows fractions that were pooled for subsequent studies. (c) Representative gel showing array DNA before reconstitution and purified chromatin and chromatosome arrays after sucrose gradient purification. The chromatin and chromatosome arrays ran above the 5 kb DNA marker band, while DNA before reconstituted ran ~ 3 kb. (d) AvaI digests of purified nucleosome arrays used to characterize the degree of saturation of the various chromatin arrays. Gels were stained with GelStarTM. (e) Example of a coomassie stained 6 – 18% SDS PAGE to control for histone octamer and CAP stoichiometry as well as their incorporation within each respective chromatin/chromatosome array. Since the ability of the type of gel limits the resolution of all 4 histone proteins and CAPs together on one gel, we show below a 20% SDS gel, showing a clearer resolution of the histones from the samples run on the gel above (6 -18% SDS gel).

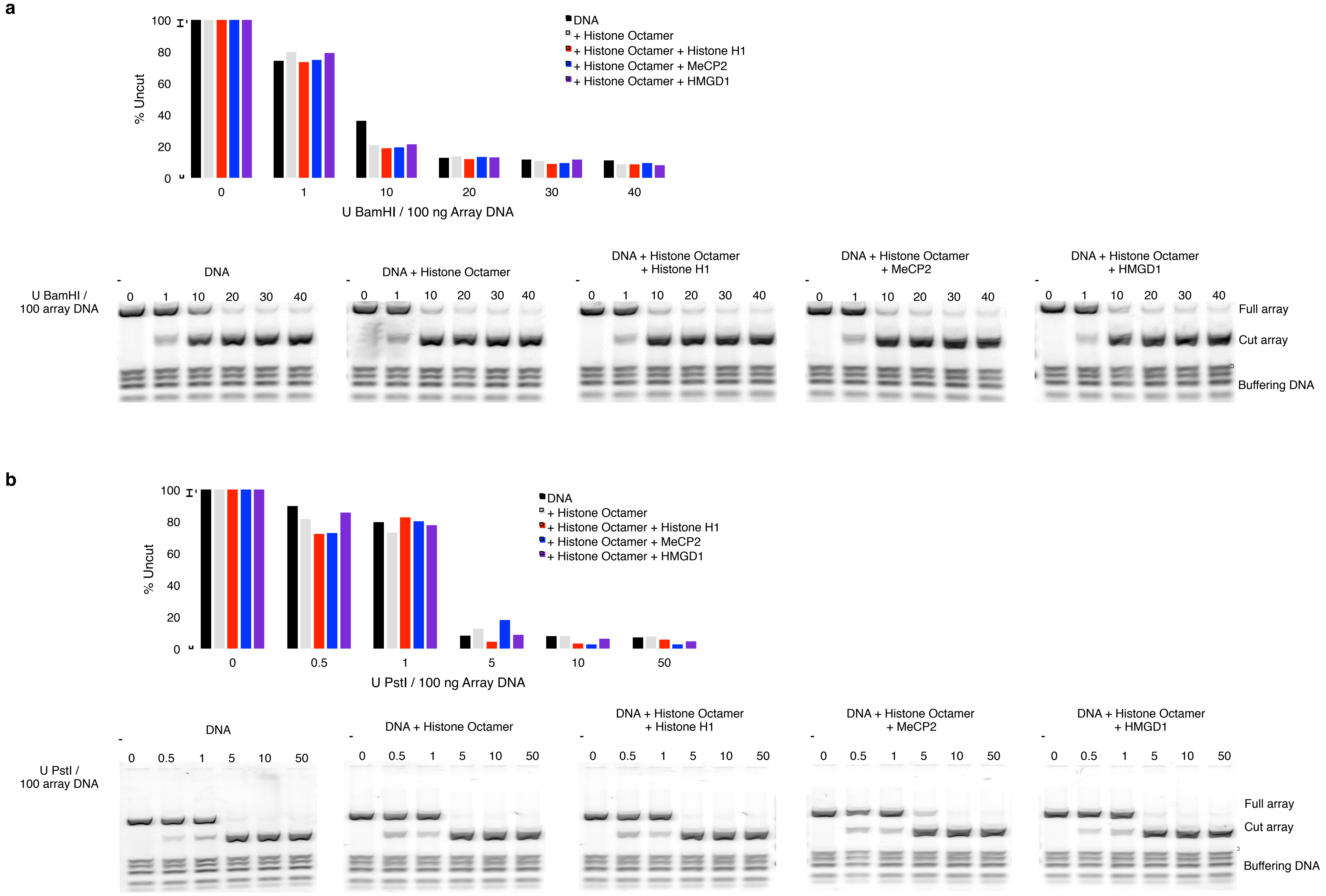

Supplemental Figure 6

Supplemental Figure 6: The presence of histone octamer or CAPs did not change restriction enzyme digest amount of the 17-mer array DNA. (a) Quantification and gel images of array DNA (still containing buffering DNA) incubated with histone octamer and CAPs in amounts equal to reconstituted arrays and digested with increasing amounts of BamHI for 1 hour or (b) PstI for 15 minutes. Restriction enzyme digestion was stopped with 1 mM EDTA pH 8.0, all samples were digested with Proteinase K and run on 1 % agarose gels with 0.2 x GelStar™. The digested array ran at ~ 1,500 bp (lower band), while the full, undigested array DNA ran at ~ 3,000 bp (top band).
